# Difference in Bone Density in Young Women With Normal Occlusion and Malocclusion

**DOI:** 10.7759/cureus.79731

**Published:** 2025-02-26

**Authors:** Haruka Hatori, Nobuyuki Udagawa, Nobuo Yoshinari, Kazuhiro Uenishi, Noriyuki Sugino, Akira Taguchi

**Affiliations:** 1 Department of Orthodontics, School of Dentistry, Matsumoto Dental University, Shiojiri, JPN; 2 Department of Biochemistry, School of Dentistry, Matsumoto Dental University, Shiojiri, JPN; 3 Department of Operative Dentistry, Endodontology, and Periodontology, School of Dentistry, Matsumoto Dental University, Shiojiri, JPN; 4 Division of Nutritional Physiology, Kagawa Nutrition University, Sakado, JPN; 5 Department of Oral and Maxillofacial Radiology, School of Dentistry, Matsumoto Dental University, Shiojiri, JPN

**Keywords:** bone density, eating, malocclusion, orthodontics, women

## Abstract

Introduction

Acquisition of higher peak bone mass in young women may prevent postmenopausal osteoporosis. Many factors, including nutritional intake, influence peak bone mass. The masticatory ability associated with nutritional intake may be lower in young women with malocclusion than in those with normal occlusion. We investigated the difference in bone mineral density (BMD) and nutritional intake between younger women with normal occlusion and malocclusion.

Methods

This study involved 45 women (mean age, 20.8 years) with normal occlusion and 49 women (mean age, 21.3 years) with malocclusion. Calcaneal BMD was measured by quantitative ultrasound. All participants completed the Food Frequency Questionnaire (FFQ). Differences in BMD, body mass index (BMI), and FFQ-obtained nutrient values between the two groups were analyzed with an independent t-test. Multiple regression analysis was also conducted to assess the association between BMD and nutrient values in both participants with normal occlusion and malocclusion.

Results

Participants with malocclusion tended to have lower BMD than those with normal occlusion (p = 0.10). The former had significantly higher vitamin A intake and lower sunlight exposure time than the latter. In the malocclusion group, participants with normal BMI had significantly higher BMD than those with both higher and lower BMI (p = 0.009 and p = 0.004, respectively). High vitamin B12 intake was also associated with higher BMD in this group (p = 0.031).

Conclusions

Malocclusion in young women influenced calcaneal BMD through nutritional intake, sunlight exposure, and BMI. Orthodontic treatment in young women with malocclusion may contribute to obtaining higher peak bone mass.

## Introduction

In Japan, there are approximately 15.9 million patients with osteoporosis, comprising 11.8 million women and 4.1 million men [1]. The rapid decline in endogenous estrogen production after menopause is associated with significant bone loss and an increased risk of fragility fracture. Fragility fractures due to osteoporosis, especially postmenopausal osteoporosis, are associated with decreased quality of life, increased risks of immobility and mortality, and higher medical care costs [2].

Peak bone mass attained at a young age is an important predictor of the onset of primary osteoporosis [3]. Factors associated with peak bone mass include age, sex, body composition, physical activity, nutrition, heredity, smoking, and lifestyle [4]. Regarding nutrition, Liu et al. [5] concluded in their meta-analysis that preventive calcium supplementation during adolescence and young adulthood, the critical period before or around the time when peak bone mass is achieved, may effectively prevent future osteoporosis. However, macronutrients such as fat and protein, micronutrients such as vitamin D, and micronutrients other than calcium and vitamin D (e.g., magnesium, vitamin C, vitamin K, and dairy products) may also be important nutrients for bone metabolism [6].

In their analysis of 13,092 Koreans aged >50 years, Hong and Kang reported that low bone mineral density (BMD) of the femoral neck was significantly associated with masticatory difficulty in older women [7]. In addition, Hong et al. [8] demonstrated significant relationships between total tooth number, which is related to masticatory function, and the probability of fracture in Korean men and women aged >65 years. Motokawa et al. [9] concluded that chewing ability was closely associated with nutrient intake, intake of different food groups, and undernutrition among 509 community-dwelling Japanese older adults. These findings imply the possibility that masticatory difficulty may influence nutritional intake, resulting in an increased risk of osteoporosis in the older population.

Patients who need orthognathic surgical correction of mandibular prognathism have masticatory dysfunction. These patients tend to have poorer masticatory function than normal individuals. In one study, masticatory efficiency was significantly greater in healthy volunteers than in patients with class II dentofacial deformity and class III dentofacial deformity in all chewing tasks tested [10]. Patients with malocclusion due to dentofacial deformity are likely to have masticatory dysfunction, resulting in poor nutritional intake and decreased peak bone mass. Little is known regarding whether long-term masticatory dysfunction due to malocclusion influences nutritional intake, leading to a decrease in peak bone mass in young women. Girls exhibit a marked increase in both standing height and bone mass during puberty, and peak BMD in both the total hip and lumbar spine is attained by the age of 18 years [11]. Bone and calcium metabolism thereafter remain constant between the ages of 19 and 30 years. Therefore, we investigated the difference in calcaneus BMD and nutritional intake between younger women near the age of 18 years with normal occlusion and malocclusion.

## Materials and methods

Participants

The normal occlusion group comprised 45 dental hygienist students (mean ± standard deviation age, 20.8 ± 1.8 years) at Matsumoto Dental University who had both normal overjet and overbite (2.0-4.0 mm) and arch length discrepancy of less than −4.0 mm. The malocclusion group comprised 48 women (age, 21.3 ± 2.4 years) with jaw deformities who visited the orthodontic clinic at Matsumoto Dental University between 2020 and 2022 and underwent surgical orthodontic treatment. The exclusion criteria were congenital abnormalities, malformations such as congenital anodontia with six or more teeth, malignant tumors, systemic syndromes, and cleft lip and palate.

Informed consent was obtained from all participants before the study. The Matsumoto Dental University Research Ethics Committee reviewed and approved the study protocol (No.: 0316). All procedures followed were in accordance with the ethical standards of the responsible committees on human experimentation (institutional and national) and with the Helsinki Declaration of 1975, as revised in 2008.

BMD measurement and estimation of nutrient values

**Figure 1:**
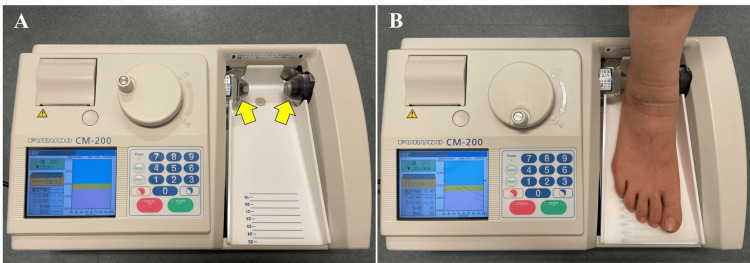
Quantitative ultrasonography. Ultrasound is transmitted and received through the opposing transducers (yellow arrows) (A), and the speed of sound (SOS) through the calcaneus is measured (B).

Right calcaneal BMD was measured as the speed of sound (m/s) in all participants using quantitative ultrasonography (CM-200; Furuno, Kobe, Japan) by one trained orthodontist (H.H.) (Figure 1). In a study by Taguchi et al. [12], the mean intra- and inter-examiner reproducibilities of the quantitative ultrasonography measurements for young Japanese adults in the Matsumoto Dental University were 3.8% and 2.1%, respectively.

Dietary nutrient intake was estimated by employing the Food Frequency Questionnaire (FFQ) for the prevention and management of osteoporosis [13]. This FFQ is especially focused on the intake of protein and calcium and their combinations with other nutritional factors. This method is based on the established semi-quantitative FFQ [14] and modified to suit the investigation of skeletal disorders. The questionnaire comprises a total of 28 food items. The participants were asked to select the grade of intake frequency for each item during the previous one month. Nutrient and energy intakes were estimated by the frequency grade and relevant coefficients determined using the Standard Tables of Food Composition in Japan [15]. In addition to completing the FFQ, the participants provided information on their status of physical activity and sunlight exposure time. The query about sunlight exposure time was divided into every day, sometimes, and rare for exposure once a day.

Statistical analysis

The categorical items of each question obtained from the FFQ were transformed and converted into continuous variables using deviation values (converted to a normal distribution with a mean of 50 and a standard deviation of 10) [16]. Because the numbers of skim milk drinkers and smokers were small (one and two, respectively), they were excluded from the analysis. A higher value for sunlight exposure, when converted to a normal distribution, indicated a lower frequency of sunlight exposure. Some nutrient values were calculated from the categorical items of each question obtained from the FFQ according to the method of Uenishi et al. [13].

Differences in age, body mass index (BMI), calcaneal BMD, categorical items of each question, exercise status, sunlight exposure, and some nutrient values calculated by the FFQ between the normal occlusion and malocclusion groups were analyzed by an independent t-test. BMI was classified into quartile hierarchies: BMI < 18.90 kg/m2 (first stratum), BMI ≤ 18.90-20.81 kg/m2 (second and third strata), and BMI > 20.81 kg/m2 (fourth stratum). In the analysis, BMI ≤ 18.90-20.81 kg/m2 was used as the standard, and these three BMI categories were treated as dummy variables.

Multiple regression analysis with the Akaike information criterion (AIC) [17] was performed using the logarithmically transformed value of calcaneal BMD as the objective variable and BMI category, occlusal status, and nutritional intakes as explanatory variables to investigate the relationship between calcaneal BMD, occlusal status, and nutrient values. The factors that determined calcaneal BMD in all participants as well as each individual participant were evaluated by multiple regression analysis. The level of statistical significance was set at a risk rate of <5% and R version 4.0.5 (R Foundation for Statistical Computing, Vienna, Austria) was used for statistical analysis.

## Results

Participants with normal occlusion had significantly greater body height (p = 0.035) and body weight (p = 0.024) than those with malocclusion (Table 1). No significant difference was observed in age, BMI, or calcaneal BMD between the groups.

**Table 1:** Differences in age, body height, body weight, BMI, and calcaneal BMD between subjects with normal occlusion and malocclusion. BMI: body mass index; BMD: bone mineral density; SD: standard deviation.

Variables	Normal occlusion group (n = 45)	Malocclusion group (n = 48)	p-value
	Mean (SD)	Mean (SD)	
Age (years)	20.8 (1.8)	21.3 (2.4)	0.306
Height (cm)	158.1 (4.7)	156.1 (4.2)	0.035
Weight (kg)	50.7 (7.2)	47.8 (4.5)	0.024
BMI (kg/m^2^)	20.2 (2.3)	19.6 (1.6)	0.140
BMD (m/s)	1547.4 (43.4)	1537.3 (25.1)	0.171

Participants with normal occlusion had significantly higher intake frequencies of butter/margarine consumption (p = 0.004) than those with malocclusion (Table 2). Conversely, participants with malocclusion had significantly higher intake frequencies of fish eggs (p = 0.010) and light-colored vegetables in the morning (p = 0.017) and a lower frequency of sunlight exposure (p = 0.020) than those with normal occlusion. The intake frequencies of liver, miso soup, oily dishes, and salty foods tended to be higher in participants with malocclusion than in those with normal occlusion. Regarding the nutrient values calculated by the FFQ, participants with malocclusion had significantly lower intake of lipids (p = 0.012) and higher intake of vitamin A (p = 0.028) than those with normal occlusion (Table 3). Consumption of iron tended to be lower in participants with normal occlusion than in those with malocclusion (p = 0.055).

**Table 2:** FFQ items converted to a normal distribution between the two groups. FFQ: food frequency questionnaire; SD: standard deviation.

Variables	Normal occlusion group	Malocclusion group	p-value
	Mean (SD)	Mean (SD)	
Fish and meat	51.4 (8.1)	49.8 (6.7)	0.308
Milk	50.7 (8.8)	49.6 (7.6)	0.503
Yogurt	51.0 (8.5)	49.9 (7.8)	0.496
Cheese	51.3 (8.2)	49.5 (7.8)	0.302
Egg	50.3 (8.9)	49.9 (7.8)	0.848
Shellfish	49.2 (8.4)	51.4 (8.2)	0.178
Squid, octopus, shrimp	49.3 (8.3)	51.2 (7.8)	0.258
Small fish	50.0 (8.4)	50.7 (7.2)	0.673
Fish eggs	48.2 (6.5)	52.4 (8.6)	0.010
Meat, processed meat	49.4 (7.4)	50.7 (9.0)	0.455
Liver	49.4 (4.9)	51.7 (7.7)	0.080
Soybeans	50.4 (8.9)	49.6 (8.4)	0.660
Natto	51.4 (9.3)	49.8 (6.9)	0.360
Potatoes	50.5 (7.8)	49.8 (8.6)	0.703
Light-colored vegetables (morning)	48.7 (5.7)	52.4 (8.6)	0.017
Green and yellow vegetables (morning)	49.8 (7.1)	51.4 (7.9)	0.317
Seaweed	49.3 (8.2)	51.1 (8.3)	0.304
Nuts	51.6 (7.7)	49.8 (6.5)	0.229
Fruit	49.9 (7.9)	50.6 (8.7)	0.697
Staple food	49.1 (8.4)	51.0 (7.8)	0.250
Size of tableware	49.4 (7.4)	50.6 (7.6)	0.436
Butter, margarine	52.7 (8.2)	48.1 (7.0)	0.004
Jam, marmalade	49.6 (7.8)	51.0 (7.9)	0.392
Miso soup	48.3 (9.1)	51.7 (8.7)	0.069
Coffee, tea	50.3 (8.3)	50.1 (9.5)	0.893
Coffee, tea (sugar)	51.1 (8.3)	50.2 (7.0)	0.592
Sweet drink	50.5 (7.1)	49.7 (8.5)	0.616
Confectionery	50.6 (7.6)	49.3 (8.7)	0.448
Cooking using oil	48.2 (7.9)	51.6 (9.3)	0.064
Amount of fat	50.9 (8.1)	50.2 (6.9)	0.632
Depth of taste	51.5 (7.9)	48.8 (8.3)	0.118
Salty food	49.2 (7.2)	51.9 (7.6)	0.081
Pickles	49.3 (7.7)	51.6 (8.4)	0.180
Alcohol consumption	50.1 (6.2)	51.3 (7.2)	0.339
Dietary supplement	50.8 (6.9)	49.9 (6.6)	0.513
Consume 3 meals a day	50.1 (6.3)	50.8 (6.7)	0.612
Sunlight exposure	49.1 (4.4)	52.1 (7.5)	0.020
Exercise	49.5 (6.5)	51.0 (6.9)	0.296

**Table 3:** Differences in nutrient values between subjects with normal occlusion and malocclusion. SD: standard deviation.

Variables	Normal occlusion group	Malocclusion group	p-value
	Mean (SD)	Mean (SD)	
Energy (kcal/d)	1431.3 (338.3)	1385.7 (317.2)	0.504
Alcohol (g/d)	10.7 (28.8)	17.5 (42.2)	0.367
Protein (g/d)	64.9 (19.1)	60.5 (16.6)	0.244
Lipid (g/d)	58.7 (12.6)	51.2 (15.5)	0.012
Carbohydrate (g/d)	164.2 (55.5)	173.6 (40.9)	0.357
Salt (g/d)	9.7 (1.4)	9.6 (1.7)	0.675
Calcium (mg/d)	322.6 (119.3)	323.3 (119.2)	0.979
Iron (mg/d)	5.4 (1.8)	6.2 (2.3)	0.055
Vitamin A (µg RE/d)	555.4 (271.0)	823.6 (776.0)	0.028
Vitamin D (µg/d)	7.6 (2.1)	8.2 (2.2)	0.171
Vitamin K (µg/d)	174.3 (144.9)	155.5 (109.3)	0.485
Vitamin B1 (mg/d)	0.7 (0.2)	0.6 (0.2)	0.890
Vitamin B2 (mg/d)	0.8 (0.3)	0.9 (0.4)	0.631
Vitamin B6 (mg/d)	0.9 (0.2)	0.9 (0.3)	0.880
Vitamin B12 (µg/d)	3.9 (2.6)	4.5 (3.7)	0.376
Folic acid (µg/d)	145.5 (50.4)	170.0 (88.5)	0.109
Vitamin C (mg/d)	48.4 (18.5)	51.2 (23.9)	0.532
Magnesium (mg/d)	148.4 (38.7)	148.5 (41.1)	0.986

In all participants, multiple regression analysis revealed an association between calcaneal BMD and intake of vitamin C and magnesium (AIC = −445.0, adjusted R2 = 0.105, p = 0.005) (Table 4). Malocclusion tended to be associated with lower calcaneal BMD (p = 0.101). Intake of vitamin C was significantly associated with higher calcaneal BMD (p = 0.002). Intake of magnesium was significantly associated with lower calcaneal BMD (p = 0.005).

**Table 4:** Factors independently associated with calcaneal BMD in all subjects. BMD: bone mineral density.

Variables	Parameter estimates	Standard error	t-value	p-value
Malocclusion	-0.007	0.004	-1.658	0.101
Vitamin C	0.004	0.001	3.174	0.002
Magnesium	-0.002	0.001	-2.892	0.005

In the normal occlusion group, the multiple regression analysis revealed an association between calcaneal BMD and intake of vitamin C and magnesium (AIC = −199.0, adjusted R2 = 0.208, p = 0.009) (Table 5). Calcaneal BMD significantly increased with increasing intake of vitamin C (p = 0.011) and decreased with increasing intake of magnesium (p = 0.002). No significant association was observed between BMI and calcaneal BMD.

**Table 5:** Factors independently associated with calcaneal BMD in subjects with normal occlusion. BMI: body mass index; BMD: bone mineral density.

Variables	Parameter estimates	Standard error	t-value	p-value
BMI < 18.90	0.001	0.011	0.087	0.931
BMI > 20.81	0.008	0.009	0.968	0.339
Vitamin C	0.006	0.002	2.683	0.011
Magnesium	-0.004	0.001	-3.328	0.002

In the malocclusion group, the multiple regression analysis revealed an association between calcaneal BMD, BMI, and intake of vitamin B12 (AIC = −264.2, adjusted R2 = 0.212, p = 0.004) (Table 6). Calcaneal BMD significantly increased with increasing intake of vitamin B12 (p = 0.031) In addition, higher BMI (>20.81 kg/m2) and lower BMI (<18.90 kg/m2) were significantly associated with lower calcaneal BMD compared with standard BMI (BMI ≤ 18.90-20.81 kg/m2).

**Table 6:** Factors independently associated with calcaneal BMD in subjects with malocclusion. BMI: body mass index; BMD: bone mineral density.

Variables	Parameter estimates	Standard error	t-value	p-value
BMI < 18.90	-0.014	0.005	-2.751	0.009
BMI > 20.81	-0.016	0.005	-2.999	0.004
Vitamin B12	0.001	0.001	2.235	0.031

## Discussion

In all participants, the presence of malocclusion tended to be associated with lower calcaneal BMD, although the association was not statistically significant. This is the first study to focus on the association of malocclusion with BMD in young women. We first hypothesized that long-term masticatory dysfunction due to malocclusion may influence nutritional intake, leading to a decrease in peak bone mass in young women. We found differences in the FFQ items related to food consumption, nutrient values, and the status of sunlight exposure. In particular, participants with malocclusion showed significantly higher intake of vitamin A than those with normal occlusion (823.6 vs. 555.4 µg RE/day). Conaway et al. [18] suggested that a high level of vitamin A is considered a risk factor for secondary osteoporosis, although Zhang et al. [19] reported in their meta-analysis that higher dietary intake of retinol and total vitamin A may slightly decrease the total fracture risk and increase the hip fracture risk. Lerner [20] recently reported that in the industrialized world, excess vitamin A has been suggested to be a risk factor for secondary osteoporosis and enhanced susceptibility to fractures. Increased amounts of vitamin A cause decreased cortical bone mass and weaker bones due to enhanced periosteal bone resorption. Excessive intake of preformed vitamin A, such as that found in supplements and animal sources (animal liver, fish liver oil, dairy products, and eggs), is associated with multisystem effects that can include bone resorption and hypercalcemia [21]. In this study, the intakes of liver and fish eggs were higher in participants with malocclusion than in those with normal occlusion, leading to a higher intake of vitamin A. Because the liver and eggs are soft, it is likely that these are easy to eat for people with malocclusion.

Participants with malocclusion had significantly less sunlight exposure than those with normal occlusion. Kurabe et al. [22] clarified the impact of orthognathic surgery on oral health-related quality of life in patients (mean age of 23.6 years) with jaw deformities. The oral health-related quality of life index included eight domains, among which the social disability domain asked, “Have you avoided going out because of problems with your teeth, mouth, or dentures?” The patients’ social disability improved after the orthognathic surgery. Patients with jaw deformity, who usually have malocclusion, are prone to loss of self-confidence, decreased social adaptation, and psychological disorders because of their conspicuous maxillofacial disharmony. Therefore, people with malocclusion are less likely to go out, resulting in a lower sunlight exposure time. Provitamin D3, which is present in the skin, is converted to vitamin D by ultraviolet irradiation. Activated vitamin D then promotes calcium absorption in the duodenum. Because short exposure to sunlight does not increase calcium absorption, this might explain why participants with malocclusion tended to have lower calcaneal BMD.

Among the participants with malocclusion, both higher and lower BMI were significantly associated with lower calcaneal BMD compared with standard BMI. However, BMI was not associated with calcaneal BMD in participants with normal occlusion. The general consensus is that people with obesity tend to have high BMD levels, making fractures less likely. However, Tanaka et al. [23] performed a cohort study of 1,614 postmenopausal Japanese women followed for 6.7 years and found that overweight/obesity and underweight were both risk factors for fractures at different sites. Postmenopausal women might have lower masticatory function than young women with normal occlusion. The detailed mechanism regarding the association between BMI and calcaneal BMD in women is still unknown, but this association may depend on masticatory function.

Calcaneal BMD significantly increased with increasing intake of vitamin C in all participants as well as participants with normal occlusion. Malmir et al. [24] concluded in their meta-analysis of observational studies that greater intake of dietary vitamin C was associated with a lower risk of hip fracture and osteoporosis as well as higher BMD at the femoral neck and lumbar spine. The results of our study are consistent with those of their study. However, calcaneal BMD was not associated with the intake of vitamin C in participants with malocclusion, although there was no significant difference in the intake of vitamin C between participants with normal occlusion and malocclusion. Increased intake of vitamin B12, not vitamin C, was significantly associated with higher calcaneal BMD in participants with malocclusion. Zhang et al. [25] reported in their meta-analysis that the serum vitamin B12 level was significantly higher in women with normal BMD than in those with postmenopausal osteoporosis. Conversely, Li and Liu [26] found no significant association between the serum vitamin B12 level and the BMD at different sites in 3,337 women aged ≥18 years in the United States. Because no studies to date have focused on the association between the vitamin B12 level and BMD in young Japanese women, a further study would be necessary to clarify this association from the viewpoint of occlusion and/or masticatory function.

Calcaneal BMD significantly decreased with increasing intake of magnesium in all participants as well as participants with normal occlusion. Kim et al. [27] reported that the level of magnesium intake per 1,000 kcal showed a significant correlation with calcaneal BMD measured as the speed of sound (r = 0.110, p < 0.05) after adjustment for covariates among 484 healthy young Korean women in their early 20s. Veronese et al. [28] analyzed 3,765 American participants (1,577 men, 2,071 women) with a mean age of 60.6 years and found that higher dietary magnesium intake had a protective effect against future osteoporotic fractures, especially in women at high risk of knee osteoarthritis. The result of our study regarding magnesium intake was consistent with these previous studies. Conversely, Nakamura et al. [29] found no significant association between magnesium intake and spinal or femoral BMD in 106 young female Japanese university students aged 19 to 25 years. In their meta-analysis, Farsinejad-Marj et al. [30] observed a positive marginally significant correlation between magnesium intake and BMD in the femoral neck and total hip. However, they did not find a significant correlation between magnesium intake and BMD in the lumbar spine. In their meta-analysis, only three studies included younger women. Because few studies have reported the effects of magnesium on peak bone mass in young women, more data are required to clarify the association between the intake of magnesium and BMD at different sites.

Our study had two main limitations. First, we included only 48 participants with malocclusion because we selected them from patients who needed surgical orthodontic treatment. This small number of participants likely influenced the results of the analysis. Second, the lifestyle and genetic factors differed between participants with normal occlusion and malocclusion. Lifestyle-related factors associated with calcaneal BMD may have differed between the dental hygienist students and patients with jaw deformities who visited the orthodontic clinic. Additionally, some genetic factors associated with jaw deformity may have influenced the results. A further study involving a large number of orthodontic patients who need or do not need surgical orthodontic treatment would be necessary to clarify our results.

## Conclusions

In the present study, young women with malocclusion tended to have lower calcaneal BMD than those with normal occlusion, although the difference did not reach statistical significance. Differences were observed in nutrition intake and factors associated with social disability, such as sunlight exposure between women with normal occlusion and malocclusion. Surgical orthodontic treatment may not only help to maintain normal BMI in young women with malocclusion but may also contribute to obtaining higher peak bone mass to some degree.
